# Clinical audit of prescribing for attention deficit hyperactivity disorder (ADHD) in children and young people services (CYPS)

**DOI:** 10.1192/bjo.2021.170

**Published:** 2021-06-18

**Authors:** Mary Parker, Elaine Martin

**Affiliations:** Tees, Esk and Wear Valleys NHS Foundation Trust

## Abstract

**Background:**

The audit aimed to assess, when 3rd and 4th line medications were prescribed for ADHD, if practice was compliant with Tees, Esk and Wear Valleys NHS Foundation Trust (TEWV) prescribing guidelines and the updated NICE Guideline NG87: Attention deficit hyperactivity disorder: diagnosis and management 2018.

**Method:**

The audit was conducted in the four Teesside Child & Adolescent community teams during April/May 2018. Each team identified all patients prescribed 3rd and 4th line ADHD medications leading to 30 responses (n = 30).

Information was collected from electronic and paper medical records using a designated audit tool compiled from the above evidence based guidelines. The data were analysed for compliance against standards using an excel spreadsheet and reviewed by the audit lead.

**Result:**

There were many areas of good practice demonstrated in the audit including diagnostic recording, pre-treatment non-medical interventions where ADHD was not severe, and use of Methylphenidate as first line medication in accordance with BNF limits. In the majority of records reviewed, there was good evidence of a variety of NICE recommended non-medication interventions which were often continued post medication initiation.

There was also very good evidence of comprehensive verbal and written information and psychoeducation including benefits and potential side effects of medication (92% verbal and 58% written).

A pre-treatment assessment was completed in all but 3 cases, 1 of which had no assessment documented and 2 cases were transferred from out of area.

Issues identified by the audit, where there was deviation from guidelines, included 4 cases where Methylphenidate was not prescribed as first line, of these, 3 were prescribed Atomoxetine due to parental choice and one was due to contraindications, suggesting patient choice was an important factor in selection of 2nd line medication.

The audit demonstrates that clinical practice had moved away from the previous guidance in NICE CG72 (to prescribe atomoxetine 2nd line) towards the prescription of Lisdexamfetamine 2nd line (75%) as reflected in the new NICE guidelines: NG87, 2018 (updated 2019).

**Conclusion:**

This audit cycle has demonstrated that use of an evidence based approach has been instrumental in improving patient care. The Audit evidenced good practice in areas such as pre-assessment, information and psychological education, initial use of Methylphenidate, use of Lisdexamfetamine 2nd line, as well as consideration of patient choice. Importantly the audit highlighted that implementation of updated NICE compliant trust guidance, followed by a planned trust-wide audit will promote continuous improvement in patient care.

